# Atorvastatin reduces β-Adrenergic dysfunction in rats with diabetic cardiomyopathy

**DOI:** 10.1371/journal.pone.0180103

**Published:** 2017-07-20

**Authors:** Aude Carillion, Sarah Feldman, Na Na, Matthieu Biais, Wassila Carpentier, Aurélie Birenbaum, Nicolas Cagnard, Xavier Loyer, Dominique Bonnefont-Rousselot, Stéphane Hatem, Bruno Riou, Julien Amour

**Affiliations:** 1 Sorbonne Universités, UPMC Univ Paris 06, UMR INSERM 1166, IHU ICAN, and Department of Anesthesiology and Critical Care Medicine, Hôpital Pitié-Salpêtrière, Assistance Publique-Hôpitaux de Paris (APHP), Paris, France; 2 Sorbonne Universités, UPMC Univ Paris 06, UMR INSERM 1166, IHU ICAN, and Department of Emergency Medicine and Surgery, Hôpital Pitié-Salpêtrière, Assistance Publique-Hôpitaux de Paris (APHP), Paris, France; 3 Sorbonne Universités, UPMC Univ Paris 06, UMR INSERM 1166, IHU ICAN, and Department of Anesthesiology and Critical Care, Université Bordeaux Segalen, Hôpital Pellegrin, Bordeaux, France; 4 Sorbonne Universités, UPMC Univ Paris 06, Post-Genomic Platform, Paris, France; 5 Sorbonne Universités, Université Paris Descartes, Bioinformatics Platform, Paris, France; 6 Sorbonne Universités, Université Paris Descartes, UMRS INSERM U970, Cardiovascular Research center, Hôpital Européen Georges Pompidou, Assistance Publique-Hôpitaux de Paris (APHP), Paris, France; 7 Sorbonne Paris Cité, Paris Descartes University, CNRS UMR8258—INSERM U1022, Faculty of Pharmacy, Department of Metabolic Biochemistry, La Pitié Salpêtrière-Charles Foix University Hospital (AP-HP), Paris, France; 8 Sorbonne Universités, UPMC Univ Paris 06, UMR INSERM 1166, IHU ICAN, Sorbonne Universités, UPMC Univ Paris 06, Hôpital Pitié-Salpêtrière, Assistance Publique-Hôpitaux de Paris (APHP), Paris, France; Max Delbruck Centrum fur Molekulare Medizin Berlin Buch, GERMANY

## Abstract

**Background:**

In the diabetic heart the β-adrenergic response is altered partly by down-regulation of the β1-adrenoceptor, reducing its positive inotropic effect and up-regulation of the β3-adrenoceptor, increasing its negative inotropic effect. Statins have clinical benefits on morbidity and mortality in diabetic patients which are attributed to their “pleiotropic” effects. The objective of our study was to investigate the role of statin treatment on β-adrenergic dysfunction in diabetic rat cardiomyocytes.

**Methods:**

β-adrenergic responses were investigated *in vivo* (echocardiography) and *ex vivo* (left ventricular papillary muscles) in healthy and streptozotocin-induced diabetic rats, who were pre-treated or not by oral atorvastatin over 15 days (50 mg.kg^-1^.day^-1^). Micro-array analysis and immunoblotting were performed in left ventricular homogenates. Data are presented as mean percentage of baseline ± SD.

**Results:**

Atorvastatin restored the impaired positive inotropic effect of β-adrenergic stimulation in diabetic hearts compared with healthy hearts both *in vivo* and *ex vivo* but did not suppress the diastolic dysfunction of diabetes. Atorvastatin changed the RNA expression of 9 genes in the β-adrenergic pathway and corrected the protein expression of β1-adrenoceptor and β1/β3-adrenoceptor ratio, and multidrug resistance protein 4 (MRP4). Nitric oxide synthase (NOS) inhibition abolished the beneficial effects of atorvastatin on the β-adrenoceptor response.

**Conclusions:**

Atorvastatin restored the positive inotropic effect of the β-adrenoceptor stimulation in diabetic cardiomyopathy. This effect is mediated by multiple modifications in expression of proteins in the β-adrenergic signaling pathway, particularly through the NOS pathway.

## Introduction

Several years after the onset of diabetes mellitus and even in well-controlled expressions of the disease, most patients develop diabetic cardiomyopathy [1–4], which may lead to diastolic heart failure [5]. Diabetes worsens perioperative prognosis partly due to more frequent cardiovascular complications [2,3]. In intensive care units, diabetic patients represent usually 30% of the population and they are known to be at increased risk of cardiac overload and mortality [4]. The β-adrenergic pathway plays a crucial role for maintaining cardiac output and the response to β-adrenergic stimulation is known to be altered in the diabetic cardiomyopathy [5,6]. The prime mechanisms of diabetic cardiomyopathy appear to be hyperglycemia and advanced generation of glycation end-products, leading to endothelial dysfunction and fibrosis and increase in inflammation and oxidative stress [7,8]. Several alterations of cardiomyocytes also contribute to cardiac dysfunction as changes in contractile proteins and impair excitation-contraction coupling by an increase in the phospholamban/sarcoplasmic reticulum calcium-ATPase ratio [9]. In addition, the positive inotropic effect of β1-adrenoceptor stimulation is reduced by a down-regulation in β1-adrenoceptor expression while the negative inotropic effect of β3-adrenoceptor stimulation is enhanced by β3-adrenoceptor overexpression. β3-adrenoceptor couple with G_i_ inhibitory G-proteins and neuronal nitric oxide (NO) synthase (NOS1), activates the phospho-inositide 3-Kinase (PI3K)/Akt pathway in the cardiomyocyte which enhances NO and cyclic guanosine monophosphate production via Rho and Ras protein activation [9,10].

Statins are competitive inhibitors of 3-hydroxy-3-methylglutaryl-coenzyme A (HMG CoA) reductase, initially developed as cholesterol-lowering drugs and a consistent reduction of heart failure and major cardiovascular adverse events has been described in large scale studies [11–14]. Several trials performed in human heart failure have shown that statin improves the left ventricular ejection fraction and exercise capacity [15]. High dose of atorvastatin (80 vs 10 mg/day) significantly decreased hospitalization for congestive heart failure [16]. In the perioperative period, statin therapy has been reported to reduce morbidity and mortality, increasingly with high-dose of statins [17,18] whatever cholesterol blood level [13,17,19], suggesting a beneficial pleiotropic cardiovascular effect [12]. In addition, pleiotropic effects of statins decrease mortality in critically ill diabetic patients [4,20,21]. In this context, statins may have a protective effect against cardiac overload.^4^ Statins have anti-inflammatory properties [12,13], reduce ischemia/reperfusion injury [22], oxidative stress and fibrosis [23], all increased in diabetes mellitus. Statins can prevent the development of cardiovascular remodeling and limit both the progression of glucose intolerance and the elevation of pro-inflammatory/fibrogenic cytokines in diabetic cardiomyopathy [24]. Many cardiac effects of statins are mediated by the preservation of the endothelial cell function but cardiomyocytes also benefit from statin treatment, through the activation of the PI3K/Akt pathway and increase in NO production from endothelial cell via endothelial NOS (NOS3) [25]. In diabetic rats, the decreased expression of NOS3 is normalized by statin treatment [24]. In contrast impact of statin treatment on altered β-adrenergic pathway signaling has not, as yet, been investigated.

The aim of this study was to test the hypothesis that atorvastatin restores at least partially the β-adrenergic signaling pathway in diabetic cardiomyopathy.

## Materials and methods

### Animals

All animals were cared according to the *European Union Guidelines for the care and use of laboratory animals* and under supervision of authorized researchers in an approved laboratory (agreement number B 75-13-08). Animal facility environmental monitoring uses a Siemens system software. This computer-based technology allows both local and remote access and control and provides continuous temperature, and photoperiod monitoring in animal holding rooms and differential air pressure alarms for biocontainment areas using sensors collecting real-time data. The Siemens system has the capability for high/low warnings and emergency alarms for selected variables set independently for each room: temperature extreme or excessive variability; relative humidity extreme; continuous, excessive or interrupted periods of light or darkness; positive differential air pressure (ABSL3 areas); RO drinking water treatment and supply system for low reservoir water levels and excessive or failed electrical supply current to water system pump motor; electrical currents to outlets powering pumps and filters for some aquatic species. Alarms outside preset limits are sent by text, email and voice to a customized, prescribed call list. The alarm provides the time/date of onset of the alarm condition, the physical site (down to the room) and specific environmental parameter in alarm. A veterinarian was present 7 days a week. Health of animals was checked every day.

The project had been approved by the relevant Animal Care Committee through the French Ministry of Higher Education and Research (Comité Régional d’Ethique en Expérimentation Animale Paris-Comité 3, Paris, France). Food and water were given *ad libitum*. Animals were fed with normal rat chow containing 10% of calories from fat, 67% from carbohydrates, and 23% from proteins, 2.791 Kcal.g^-1^ (A04-10, SAFE, Augy, France). Six-week-old male Wistar rats (Janvier, Le Genest-St Isle, France) were divided into a healthy group and a diabetic group. Diabetes mellitus was induced by streptozotocin (65 mg.kg^-1^, single intravenous bolus; Sigma-Aldrich, L’Isle d’Abeau Chesnes, France) as previously described [6]. After 6 weeks, rats from healthy or diabetic groups were assigned to orally receive over 15 days either vehicle or atorvastatin (50 mg.kg^-1^ day-1 per os, Pfizer, Paris, France) [26]. Diabetes mellitus was assessed by major glycosuria from 8 days after streptozotocin injection. Glucose and bicarbonate levels were determined on a blood sample withdrawn immediately after heart removing to ensure diabetes (*i*.*e*. blood glucose level > 35 mmol.L^-1^, *i*.*e*. 631 mg.dL^-1^) without acidosis. Mild diabetic rats with blood glucose level lower than 35 mmol.L^-1^ despite streptozotocin injection were excluded. Total cholesterol, LDL cholesterol and triglycerides blood levels were also measured from the same samples by automated enzymatic methods.

### Echocardiography

Transthoracic stress echocardiography was performed with a 8-14MHz linear probe and a Vivid™ 7 cardiovascular ultrasound system (General Electric, Aulnay-sous-Bois, France) on rats maintained under light general anesthesia with isoflurane (1–2%). Left ventricular systolic and diastolic diameters, left ventricular ejection fraction (LVEF) and left ventricular shortening fraction (LVSF) were determined on parasternal long- and short-axis views. Cardiac filling pressures and diastolic function were evaluated by Doppler imaging on an apical window. Left ventricular trans-mitral filling flow was described by early filling velocity (the E wave), and atrial filling velocity (the A wave). High cardiac frequency in small animals often results in E and A wave fusion. The end-diastolic left ventricle pressure was estimated by the E/Ea ratio between the E wave and the systolic velocity of lateral mitral annulus Ea, measured by Doppler tissue imaging. The diastolic function was assessed by the trans-mitral filling flow with the isovolumetric relaxation time (IRVT) and the deceleration time of the E wave. All measurements were performed in duplicate and after stabilization of the heart rate, before and 6 min after β-adrenergic stimulation by isoproterenol (10 μg.kg^-1^.min^-1^, continuously intravenous infused).

### Isolated left ventricle papillary muscle

Shortly after induction of general anesthesia with pentobarbital, the heart was removed *in bloc*, dissected and weighed. The left ventricular papillary muscles were carefully excised and suspended vertically in a Krebs-Henseleit bicarbonate buffer solution at 29°C and bubbled with 95% O2 and 5% CO_2_ as previously described [6]. The papillary muscles were stimulated at 12 Hz for 60-min stabilization period at the initial muscle length at the apex of length–active isometric tension curve (L_max_). The extracellular concentration of Ca^2+^ was decreased for all measurements from 2.5 mmol.L^-1^ to 0.5 mmol.L^-1^ because rat myocardial contractility is nearly maximal at 2.5 mmol.L^-1^. A sequence of three twitches was applied to each muscle to determine the value of usual parameters: the maximum unloaded shortening velocity (V_max_) and the maximum isometric active force (AF), the time to peak force (TPF), and the time to peak shortening (TPS) [6]. The active force AF was normalized for cross-sectional area (calculated from the length and weight of papillary muscle, assuming a density of 1). β-adrenoceptor stimulation was induced with cumulative concentrations of isoproterenol (10^−8^ to 10^−4^ mol.L^-1^), a non-selective β-adrenoceptor agonist, in the presence of phentolamine (10^−6^ mol.L^-1^). The effect of isoproterenol was expressed by the percentage of baseline value of the maximal effect of isoproterenol on AF and V_max_ (_max_Eff) and the concentration of isoproterenol producing 50% of the maximal effect (C_50_) [6].

### Microarray analysis of whole-genome gene expression

Left ventricles were harvested immediately after left ventricular papillary muscles had been excised and used for gene expression and immunoblotting (*vide infra*). Three biological replicates were used for the gene expression profiling, *i*.*e*. samples from three animals of each group, healthy or diabetic rats and treated or not by atorvastatin. Total RNA from frozen left ventricle samples was extracted with an RNeasy extraction kit (Qiagen Inc., Chatsworth, CA) following the protocol and quantified using NanoDrop 1000 (Thermo Fisher Scientific, Illkirch, France). RNA purity and integrity were assessed on a RNA NanoChip (Bioanalyzer Agilent Technologies, Santa Clara, CA). Only the samples with RNA integrity number (RIN) > 8 were used for gene expression profiling. RNA was prepared with the Illumina® TotalPrep RNA Amplification Kit (Applied Biosystems, Villebon-sur-Yvette, France) with reverse transcription and labeling of newly transcribed RNA with biotin-16-UTP. For each sample the labeled cRNA was hybridized according to the protocol on a microarray using Illumina Rat Ref– 12 expression Beadchip. RNA molecules matched with the corresponding probe between the 22 523 probes lining on the chip. After washing, the chip was exposed to a laser and, using the Illumina Iscan (Illumina, Inc, San Diego, CA), we quantified the intensity of the emitted signal in each location of the chip, measuring the relative abundance of the RNA targeted by the local probe.

Data were normalized with the quantile function of BeadStudio Software (Illumina, Inc). The quality of the signal was assessed by the signal to noise ratio and the number of normalized signals that differed from the background. First, biological replicates were compared two by two, with a required correlation coefficient > 0.98 to detect procedure abnormalities. Second, we conducted an unsupervised analysis with hierarchical clustering using the Spearman correlation similarity measure and ward linkage algorithm to confirm the correct identity of samples by expression similarities. The expression of the 22 523 targeted genes was then analyzed in the four groups. A difference of 1.5 fold in gene expression and a *p* value <0.05 between two groups was considered as significant. Data were subsequently subjected to Ingenuity pathway analysis (IPA) (Ingenuity Systems Inc., Redwood City, CA) to model relationships among genes and proteins and to construct putative pathways and relevant biological processes.

### Protein expression by immunoblotting

The left ventricles were removed and frozen in liquid nitrogen. Total proteins were extracted in a Triton 1% buffer with anti-protease inhibitor (Sigma–Aldrich). All protein concentrations were determined using the Bradford reagent (Bio-Rad, Marnes-La-Coquette, France). After denaturation in Laemmli buffer, a fixed amount of mixed proteins was loaded in each lane of a 12% sodium dodecyl sulfate polycracylamide gel electrophoresis (SDS-PAGE). Proteins were separated by electrophoresis in a migration buffer then transferred to a nitrocellulose membrane (Hybond, GE Healthcare, Vélizy, France). After saturation in milk, each membrane was incubated overnight at 4°C with primary antibodies: respectively anti-β_1_-adrenoceptor (1:1000, Affinity Bioreagents, Saint-Quentin en Yvelines, France), anti-β3-adrenoceptor (1:1000, Santa Cruz Biotechnology, Le Perray en Yvelines, France), anti-multidrug resistance protein 4 (MRP4) (1:200, Abcam, Paris, France), anti-NOS1 (1:500, Affinity BioReagents) and anti-NOS3 (1:1000, Cell Signaling, Paris, France). The day after, membranes were washed with Tris-buffer saline Tween and incubated with appropriate secondary antibody. Relative quantification of targeted protein was achieved by fluorescence recording on EthanDIGE reader with an ECL^®^ detection system (GE Healthcare, Vélizy, France). The β_1_-adrenoceptor proteins were detected at 55 kDa, β_3_-adrenoceptor at 44 kDa, and MRP4 at 150 kDa, NOS1 at 155 kDa, and NOS3 at 140 kDa. All Western blot experiments were quantified using Image J software (NIH, Bethesda, MA) and normalized versus GAPDH (37kDa) (NOS1, NOS 3, MRP4) or total proteins using Ponceau S solution (β_1_- and β_3_-adrenoceptors) to ensure no variation in protein gel loading.

### Nitric oxide synthase blockade

An additional group of diabetic rats (treated with atorvastatin) was exposed to N^*G*^-nitro-L-arginine methylester (L-NAME, 10^−5^ mol.L^-1^, Sigma-Aldrich), an unspecific NO synthase inhibitor, as previously described [6]. β-adrenoceptor stimulation was induced with cumulative concentrations of isoproterenol in isolated left ventricular muscle as described above.

### Statistical analysis

Data are expressed as mean ± SD. Means were compared using Student’s t test or one-way analysis of variance with post-hoc test Newman-Keuls. We used absolute values to compare baseline characteristics and delta percent changes from baseline to compare the pharmacological effects since SD of delta percent changes represents the variation of the pharmacological effect measured whereas the SD of absolute values mainly reflects inter-individual variation. Moreover, some variables were expected to significantly differ at baseline between groups. Concentration response curves were determined by fitting the data to the Hill sigmoid pharmacological model according to the following equation: Eff_o_ = _max_Eff. (1 + (C_50_. C^-1^)^n^)^- 1^ in which Eff_o_ is the observed effect, _max_Eff the maximum effect, *C*_50_ the concentration that results in 50% of _max_Eff, *C* the studied concentration, and *n* the Hill coefficient. Iterative nonlinear regression curve fitting was used to obtain the best fit (Matlab 1.2c software; The MathWorks, South Natick, MA). The main endpoint of our study was the *max*Eff of the concentration-response curve of AF with isoproterenol. Assuming a value of _max_Eff of 179 ± 15% in the control group [6], an alpha risk of 0.05 and a beta risk of 0.20, we determined that a sample size of at least n = 7 papillary muscle per group would enable us to detect a 15% change in _max_Eff (PASS 11 software, Statistical Solutions Ltd.).

All *p* values were two-tailed and a *p*<0.05 was considered as significant. Statistical analysis was performed using NCSS 7.0 software (Statistical solutions Ltd., Cork, Ireland).

## Results

We studied 48 healthy and 56 diabetic rats. Despite streptozotocin injection, 3 rats were excluded for insufficient blood glucose level, 2 in the statin diabetic group and 1 in the untreated diabetic group. Physical characteristics, blood chemistry results, baseline echocardiography and papillary muscle variables are shown in Table 1. All diabetic rats had significant lower body weight and heart weight than healthy rats. The heart weight/body weight ratio was slightly increased in diabetic untreated rats but corrected by atorvastatin. Atorvastatin had no significant effect on body or heart weight in healthy rats. Blood glucose level increased more than 4-fold in diabetic rats compared to healthy rats. Total and LDL cholesterol blood level were increased in diabetic rats compared to healthy rats. Atorvastatin did not significantly modify elevated blood glucose or cholesterol level in statin diabetic rats. Serum bicarbonate levels were not significantly different between groups, indicating that no ketoacidosis occurred.

**Table 1:** General characteristics of healthy and diabetic treated (*atorvastatin*, 50 mg. kg^-1^.day^-1^) and control rats.

	Healthy untreated	Healthy statin	Diabetic untreated	Diabetic statin
**General Characteristics (Number of rats)**	23	25	20	33
**Body weight (g)**	380 ± 66	405 ± 81	240 ± 44*	238 ± 57*‡
**Heart weight (mg)**	828 ± 173	850± 174	575 ± 120*	559 ± 160*‡
**Heart weight/ body weight (mg.g**^**-1**^**)**	2.2 ± 0.3	2.1 ± 0.2	2.4 ± 0.3*	2.3 ± 0.2†
**Blood glucose (mM)**	9.8 ± 1.2	9.6 ± 1.7	41.7 ± 5*	44.1 ± 8.1*‡
**Plasma bicarbonate (mM)**	28 ± 5	27 ± 5	28 ± 7	27 ± 4
**Lipid measurement (number of rats)**	6	6	6	6
**Total cholesterol (g.L**^**-1**^**)**	0.50 ± 0.03	0.43 ± 0.23	0.69 ± 0.12*	0.67 ± 0.13*
**Triglycerides (g.L**^**-1**^**)**	0.96 ± 0.21	1.03 ± 0.32	0.55 ± 0.29	0.87 ± 0.49
**LDL cholesterol (g.L**^**-1**^**)**	0.06 ± 0.01	0.06 ± 0.02	0.14 ± 0.03*	0.12 ± 0.03*†
**Echocardiography (number of rats)**	18	18	17	16
**Heart rate (beats.min**^**-1**^**)**	352 ± 34	342 ± 18	309 ± 26*	298 ± 37*
**Left ventricle ejection fraction (%)**	83 ± 8	84 ± 7	87 ± 5	79 ± 7†
**Left ventricle shortening fraction (%)**	49 ± 7	49 ± 9	52 ± 7	44 ± 6*†
**E (ms)**	0.92 ± 0.17	0.88 ± 0.14	0.92 ± 0.17	0.89 ± 0.11
**IVRT (ms)**	18 ± 5	19 ± 6	31 ± 9*	24 ± 7*†‡
**DT (ms)**	41 ± 11	37 ± 10	36 ± 9	43 ± 11
**E/E**_**a**_	17 ± 4	17 ± 4	21 ± 6*	20 ± 5
**Mechanical Properties (number of muscles)**	8	8	8	15
**V**_**max**_ **(L**_**max**_**.s**^**-1**^**)**	3.40±0.27	3.32±0.65	2.70±0.25*	.51±0.31*‡
**AF/s (mN.mm**^**-2**^**)**	65±22	64±19	67±13	66±26
**TPS (ms)**	165±8	175±11	222±14*	205±17*†‡
**TPF (ms)**	144±10	167±16*†	203±18*	184±23*†

Data are mean ± SD

*: *p*<0.05 versus untreated healthy group

†: *p*<0.05 between statin and untreated rats in each group healthy or diabetic rats

‡: *p*<0.05 between healthy statin rats and diabetic statin rats. IVRT = Isovolumic relaxation time; E = peak velocity of early mitral flow; DT = deceleration time of E wave, E/Ea = E peak velocity of early mitral flow/Ea early diastolic velocity of lateral mitral annulus ratio; V_max_ = maximal unloading isotonic shortening velocity (*L*max s-1); AF/s = active force normalized per cross-sectional area during isometric contraction; TPF = time to peak force; TPS = time to peak shortening.

During basal echocardiography, the heart rate was slower in the diabetic rats compared with healthy rats. The LVEF and the LVSF were slightly reduced in diabetic statin rats compared to other groups but remained in normal range. The IVRT was increased in diabetic rats compared to control rats and partially restored by atorvastatin in diabetic group. The *E*/*E*a ratio was slightly increased in untreated diabetic group compared with healthy group (Table 1).

In isolated papillary muscles, during basal studies AF was preserved in diabetic rats whereas V_max_ was reduced in diabetic rats and not corrected by atorvastatin. Both TPF and TPS were increased in diabetic rats and not corrected by atorvastatin (Table 1).

### Effect of atorvastatin on in vivo response to β-adrenergic stimulation

Echocardiographic parameters of *in vivo* responses to isoproterenol infusion are shown in Table 2, expressed in percentage of baseline. In additional data, we show the results expressed in absolute values (S1 Table). SD of delta percent changes represents the variation of the pharmacological effect we are measuring, whereas the SD of absolute values mainly reflects inter-individual differences. Thus, the absolute values are not adapted to investigate the pharmacological effect of isoproterenol. The heart rate was moderately increased in all groups after isoproterenol. The inotropic response was reduced in diabetic rats compared to healthy rats with reduced increase in LVEF and LVSF. Atorvastatin improved this inotropic response in diabetic rats, returning to that of healthy rats. Atorvastatin had no effect on response to β-adrenergic stimulation in healthy rats.

**Table 2:** Echocardiographic measurement of inotropic and lusitropic response to the β-adrenoceptor stimulation in healthy and diabetic rats pretreated or not with atorvastatin.

Percentage of baseline value	Healthy untreated (n = 10)	Healthy statin (n = 10)	Diabetic untreated (n = 9)	Diabetic statin (n = 8)
**Heart rate**	118 ± 17^▪^	110 ± 40^▪^	130 ± 17^▪^	114 ± 18^▪^
**LVEF**	123 ± 13^▪^	120 ± 9^▪^	108 ± 6^▪^	128 ± 11^▪^*†
**LVSF**	160 ± 19^▪^	157 ± 25^▪^	125 ± 16^▪^	167 ± 30^▪^*†
**IVRT**	94 ± 21	61 ± 14 ^▪^†	71 ± 34	82 ± 25
**DT**	99 ± 24	102 ± 35	100 ± 28	91 ± 40
**E/Ea**	102 ± 28	121 ± 47	84 ± 38	99 ± 21

Data are mean percentages of baseline values ± SD.

^▪^ *p* < 0.05 vs. baseline value

*: *p*<0.05 versus untreated healthy group

†: *p*<0.05 between statin and untreated rats in each group healthy or diabetic rats

‡: *p*<0.05 between healthy statin rats and diabetic statin rats. LVEF = left ventricular ejection fraction; LVSF = left ventricular shortening fraction; IVRT = isovolumic relaxation time; E = peak velocity of early mitral flow; DT = deceleration time of E wave, E/Ea = E peak velocity of early mitral flow/Ea early diastolic velocity of lateral mitral annulus ratio.

### Effect of atorvastatin on *ex vivo* response to β-adrenergic stimulation

**Fig 1:**
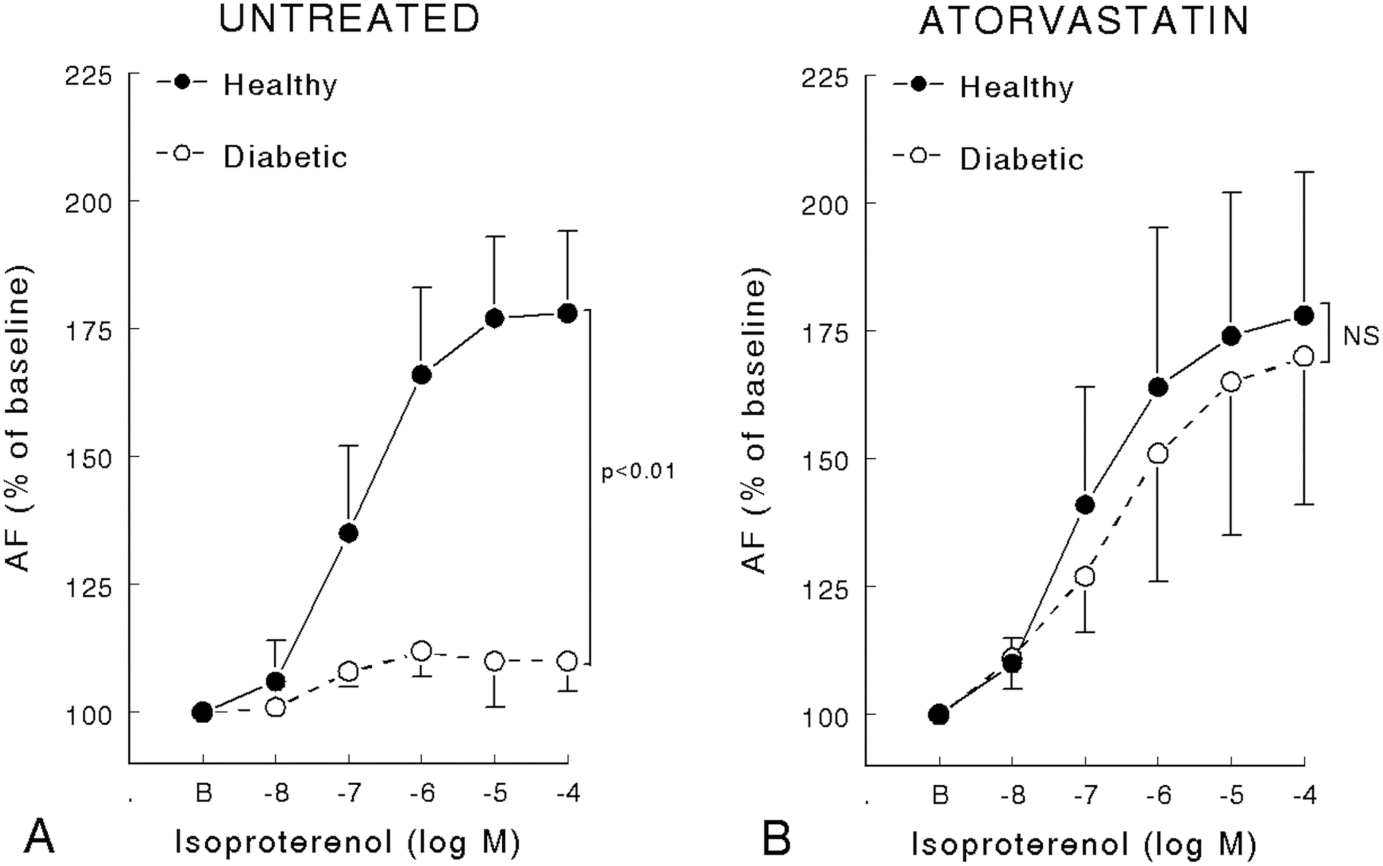
**Active force (% of baseline value) variation of left ventricle papillary muscle of healthy and diabetic rats (Panel A) or pretreated (Panel B) with atorvastatin (50 mg kg-1.day-1) during 15 days (8 rats per group) on in vitro inotropic response to isoproterenol.** Data are expressed as mean ± SD. *P* values refers to comparison between healthy and diabetic rats. NS = non-significant.

The main characteristics of the inotropic response of left ventricle papillary muscle of healthy and diabetic rats statin pretreated or not are summarized in Fig 1 and Table 3. _max_Eff on both AF and V_max_ was reduced in diabetic untreated rats compared with healthy rats and partially corrected by atorvastatin. C_50_ on both AF and V_max_ was reduced in diabetic rats, treated or non-treated with atorvastatin. Atorvastatin had no effect on healthy rats but increased _max_Eff on both AF and V_max_ in diabetic rats (Table 3). Absolute values are provided (S2 and S3 Tables) but the SD of absolute values mainly reflects interindividual differences whereas SD of delta percent changes represents the variation of the pharmacological effect we are measuring.

**Table 3:** Comparison of inotropic response to β-adrenergic stimulation of left ventricle papillary muscles of healthy or diabetic rats, pretreated or not with atorvastatin.

Isoproterenol	Healthy untreated (n = 8)	Healthy statin (n = 8)	Diabetic untreated rats (n = 8)	Diabetic statin (n = 8)				
	V_max_	AF/s	V_max_	AF/s	V_max_	AF/s	V_max_	AF/s
_**max**_**Eff (% baseline value)**	181±11*	184±22*	194±30*	177±22*	128±7*	112±15*^†^	174±27*^‡^	158±34*^†^^‡^
**C**_**50**_ **(**μ**M)**	0.16±0.15	0.20±0.15	0.91±1.14	0.81±1.15	0.07±0.06	0.07±0.05	0.09±0.08	0.28±0.34

Data are mean ± SD

*: *p*<0.05 versus untreated healthy group

†: *p*<0.05 between statin and untreated rats in each group healthy or diabetic rats

‡: *p*<0.05 between healthy statin rats and diabetic statin rats. V_max_ = maximal unloading isotonic shortening velocity; AF/s = active force normalized per cross-sectional area during isometric contraction; _max_Eff = maximal effect of isoproterenol on AF as percentage of baseline value; C_50_ = concentration of isoproterenol producing 50% of _max_Eff.

### Transcriptomic effect of atorvastatin

**Fig 2:**
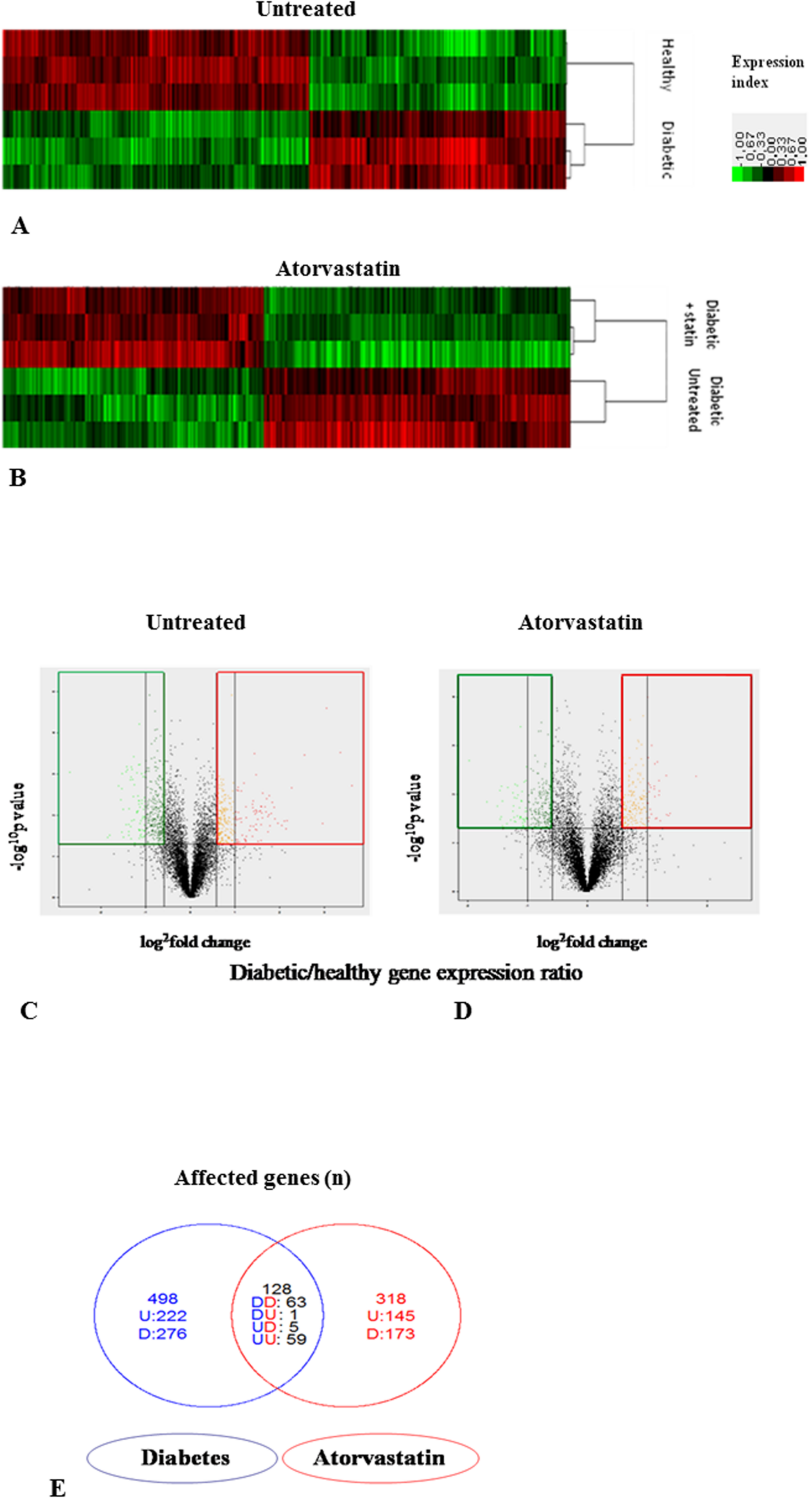
Effects of atorvastatin on the transcriptome of left ventricles of healthy or diabetic rats. Panel A-B Heat Map of RNA expression profiles in diabetic versus healthy left ventricles (Panel A) or in statin diabetic versus untreated diabetic left ventricles (Panel B); Color scale indicate relative expression ratio for each gene in diabetic versus healthy left ventricle (Panel A) or in statin versus untreated diabetic left ventricle. Panel C-D Volcano Plot for the modification of genes expression by diabetes in heart ventricle (Panel C) and by atorvastatin in diabetic left ventricle (Panel D). The vertical axis represents the *p* value (-log^10^ *p* value) and the horizontal axis range the fold change (log^2^ ratio) between diabetic and healthy left ventricles (Panel C) or statin diabetic versus untreated diabetic left ventricles (Panel D) (by t-test). Genes in the area delimited in red have a fold change greater than 1.5 with a *p* value < 0.05. Genes in the area delimited in green have a fold change greater than -1.5 (ratio <0.67) with a *p* value < 0.05. Panel E Venn diagram representing the differently expressed genes in diabetic versus healthy left ventricles in blue and in statin diabetic versus untreated diabetic left ventricles in red (*p*<0.05). D is for down-regulation in diabetic versus healthy left ventricles, U for up-regulation. The overlapping part represents the genes modified by diabetes as well as statin, with up- or down-regulation for each comparison.

The main findings of the microarray analysis on the transcriptome of left ventricles of healthy and diabetic rats with and without statin pretreatment are summarized in Fig 2. The RNA expression profiles revealed numerous genes differently expressed in diabetic versus healthy left ventricles (Fig 2A) with a strong effect of the disease on left ventricular gene expression. 626 genes were significantly differently expressed (fold change ≥ 1.5 and p-value <0.05) in diabetic versus healthy left ventricles (Fig 2C). The mRNA of 286 genes was more abundant in diabetic versus healthy left ventricles whereas the mRNA of 340 genes was less abundant. Atorvastatin also significantly modified gene expression in the left ventricles of diabetic rats but within a less important magnitude than that of diabetes itself (Fig 2B). 446 genes were significantly differently expressed (fold change ≥1.5 and *p*<0.05) in statin treated diabetic versus untreated diabetic left ventricles (Fig 2D). The mRNA of 205 genes was more abundant in diabetic statin diabetic versus untreated diabetic left ventricles whereas the mRNA of 241 genes was less abundant. The effect of diabetes and atorvastatin on gene expression was opposite in 6 genes (Fig 2E). Down-regulation in diabetes compared with healthy and up-regulation by treatment in diabetes was observed only for eNOS. Up-regulation in diabetes compared to healthy and down-regulation following treatment in diabetes was observed for 5 genes: NUDT4, GSMT5, predicted NUBPL, predicted RGD1306157 and LOC362480.

Only 8 probes found differences between statin healthy and untreated healthy left ventricles; none of these genes were involved in cardiac contraction or β–adrenergic signaling (data not shown).

### Transcriptomic effect of atorvastatin on β-adrenergic signaling pathway

Among genes involved in β-adrenergic signaling, micro-array analysis identified 9 genes differently expressed in diabetic left ventricles compared with diabetic left ventricles treated by atorvastatin (Table 4). All except phosphodiesterase 2A (PDE2A) and Ras-related associated with diabetes (RRAD) were also differently expressed in diabetic versus healthy left ventricles. NOS3 mRNA was 3.5 fold more abundant in diabetic left ventricles treated by atorvastatin than in non-treated diabetic left ventricles, whereas it was reduced 2 fold in diabetic versus healthy left ventricles. This was the only gene where we found an adverse effect of diabetes and atorvastatin. Adenylate cyclase 4 mRNA, arrestin domain containing 1 (ARRDC1), calcium/calmodulin-dependent protein kinase II inhibitor 1 (Camk2n1) and phospholipase A2GIVB mRNAs were respectively 1.9, 1.6, 1.7 and 1.7 fold more abundant in diabetic left ventricles treated by atorvastatin than in non-treated diabetic left ventricles.

All were already respectively 1.6, 1.4, 1.6, 1.5 fold more abundant than in healthy left ventricles. Troponin C1 and phospholipase A2G5 mRNAs were respectively 2.0 and 2.1 fold less abundant in diabetic left ventricles treated with atorvastatin than in non-treated diabetic left ventricles, where they were already respectively 2.0 and 1.9 fold less abundant than in healthy left ventricles. PDE2A mRNA was 1.6 fold more abundant in diabetic left ventricles treated by atorvastatin than in non-treated diabetic left ventricles, where it was not different from healthy left ventricles. RRAD mRNA was 2.0 fold less abundant in diabetic left ventricles treated by atorvastatin than in non-treated diabetic left ventricles, whereas it was not modified in diabetic versus healthy left ventricles.

**Table 4:** Expression analysis of genes significantly modified by atorvastatin in diabetic left ventricle and involved in β-adrenergic signaling.

Function	Gene	Gene expression fold change	
		Diabetic treated/diabetic control	Diabetic/healthy
**cAMP production**	Adenylate Cyclase 4 (ADCY4)	+ 1.9	+ 1.6*
**cAMP degradation**	Phosphodiesterase 2A (PDE2A)	+ 1.6	+ 1.0
**Contraction effectors**	Troponin C type 1 (TNNC1)	- 2.0	- 2.0 *
	Myosin binding protein H-like (MYBPHL)	+ 2.8	- 2.5
	Myosin, heavy polypeptide 7Bβ (MYH7B) (predicted)	+ 2.6	+ 1.3*
**Regulation pathways**	Nitric Oxide synthase 3 (NOS3) (endothelial)	+ 3.5	- 2.0*
	Arrestin domain containing 1 (ARRDC1) (predicted)	+ 1.6	+ 1.4*
	Ras-related associated with diabetes (RRAD)	- 2.0	+ 1.0
	Rho-associated kinase 2 (ROCK2)	+1.9	+1.7*
	Calcium/calmodulin-dependent protein kinase II inhibitor 1 (CAMK2N1)	+ 1.7	+ 1.6*
	Phospholipase A2G4B (PLA2G4B) (predicted)	+ 1.7	+ 1.5*
	Phospholipase A2G5 (PLA2G5)	- 2.1	- 1.9 *

Data are mean. A gene expression fold change over ±1.5 was considered as significant.

*: *p*<0.05 between Diabetic Untreated versus Healthy Untreated rats left ventricles.

### Expressions of proteins involved in β-adrenergic signaling

**Fig 3:**
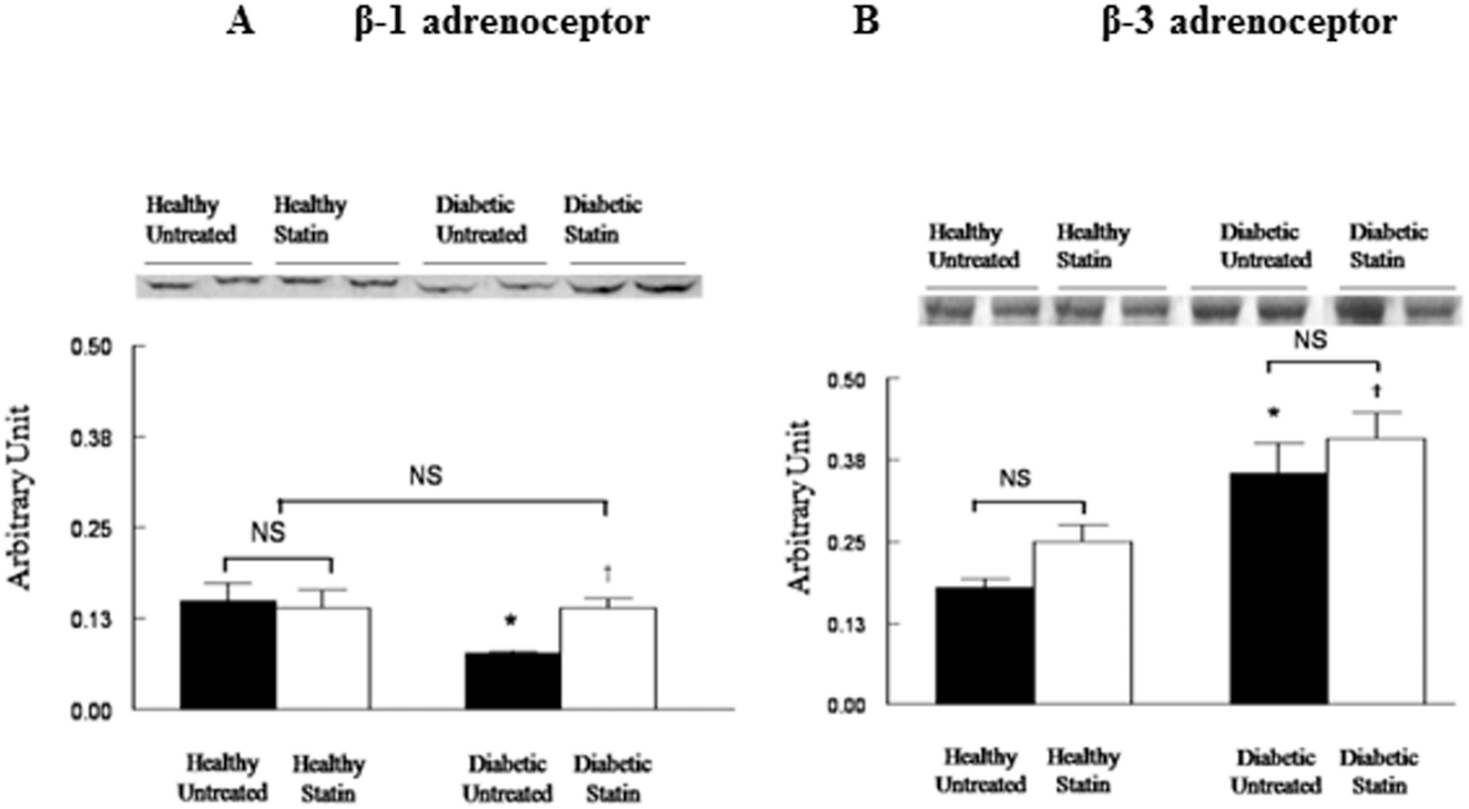
**Representative western blot and densitometric data reflecting protein expressions of β1-adrenoceptor (Panel A) and β3-adrenoceptor (Panel B) in left ventricles homogenates of healthy or diabetic rats, treated or not by atorvastatin (50 mg kg-1.day-1) during 15 days.** Western blot experiments were normalized using proteins using Ponceau S solution. Data are means ± SD (n = 4 to 9). *: *p*<0.05 versus healthy untreated rats; †: *p*<0.05 diabetic statin versus diabetic untreated rats.

**Fig 4:**
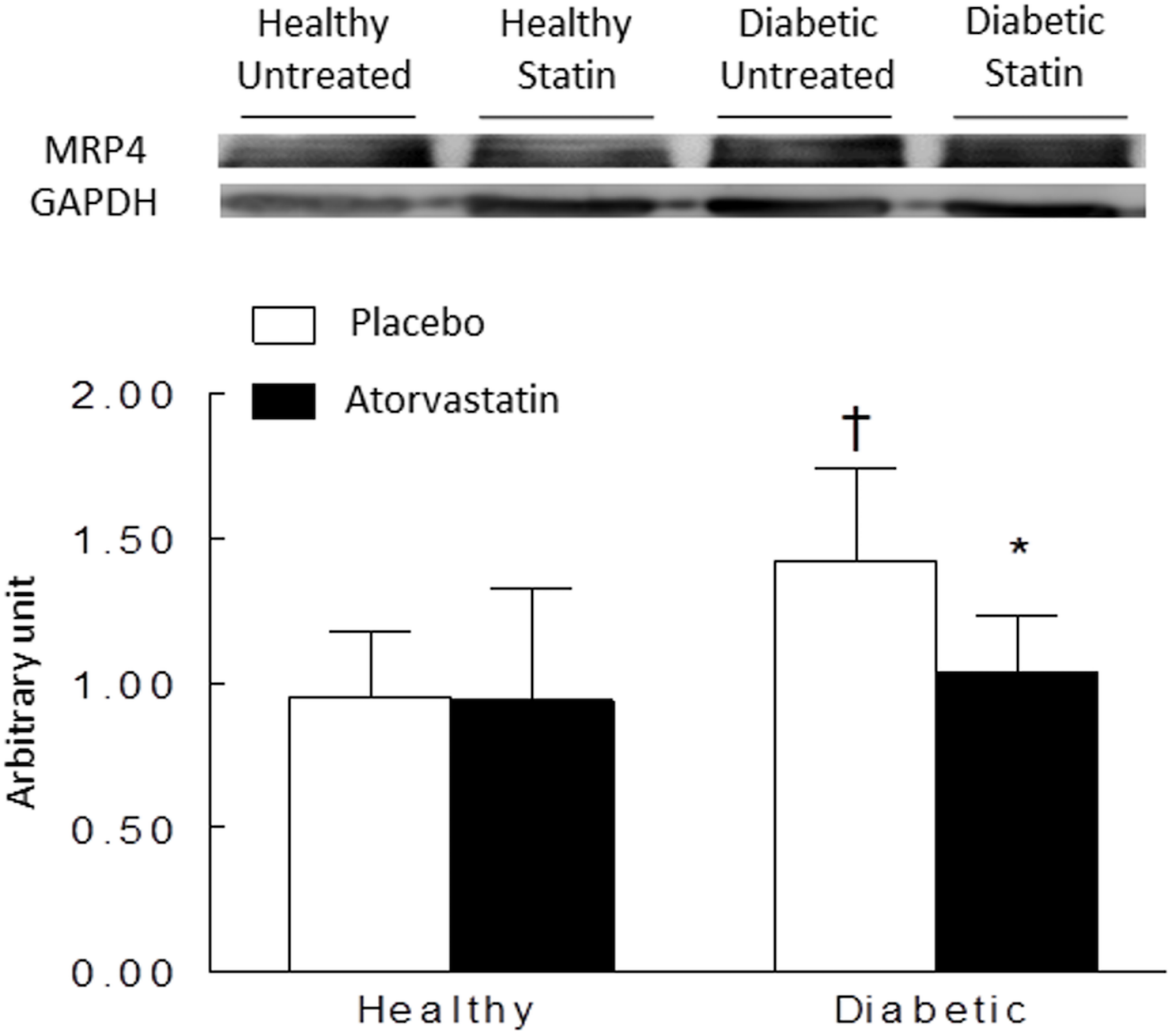
Representative Western Blot and densitometric data reflecting protein expressions of multidrug resistance protein 4 (MRP4) in left ventricles homogenates of healthy or diabetic rats, treated or not by atorvastatin (50 mg kg-1.day-1) during 15 days. Western blot experiments were normalized using GAPDH (37kDa). Data are means ± SD (n = 6). *: *p*<0.05 versus healthy untreated rats; †: *p*<0.05 diabetic statin versus diabetic untreated rats.

Protein expressions in left ventricles of healthy or diabetic rats, treated or not by atorvastatin (6 hearts in each group) are represented in Fig 3. The expression of β1-adrenoceptor was reduced in diabetic untreated rats compared with healthy rats but corrected by atorvastatin (Fig 3A). In contrast, β3-adrenoceptor increased in both untreated and atorvastatin diabetic rats compared with healthy rats (Fig 3B). There was no significant difference in diabetic group with our without pre-treatment with atorvastatin. Atorvastatin did not modify β3-adrenoceptor expressions in healthy rats left ventricles. The cAMP transporter MPR4 was increased in diabetic untreated rats compared to healthy rats and corrected by atorvastatin (Fig 4). No significant changes were observed for NOS1 and NOS3 (data not shown).

### NO synthase blockade

**Fig 5:**
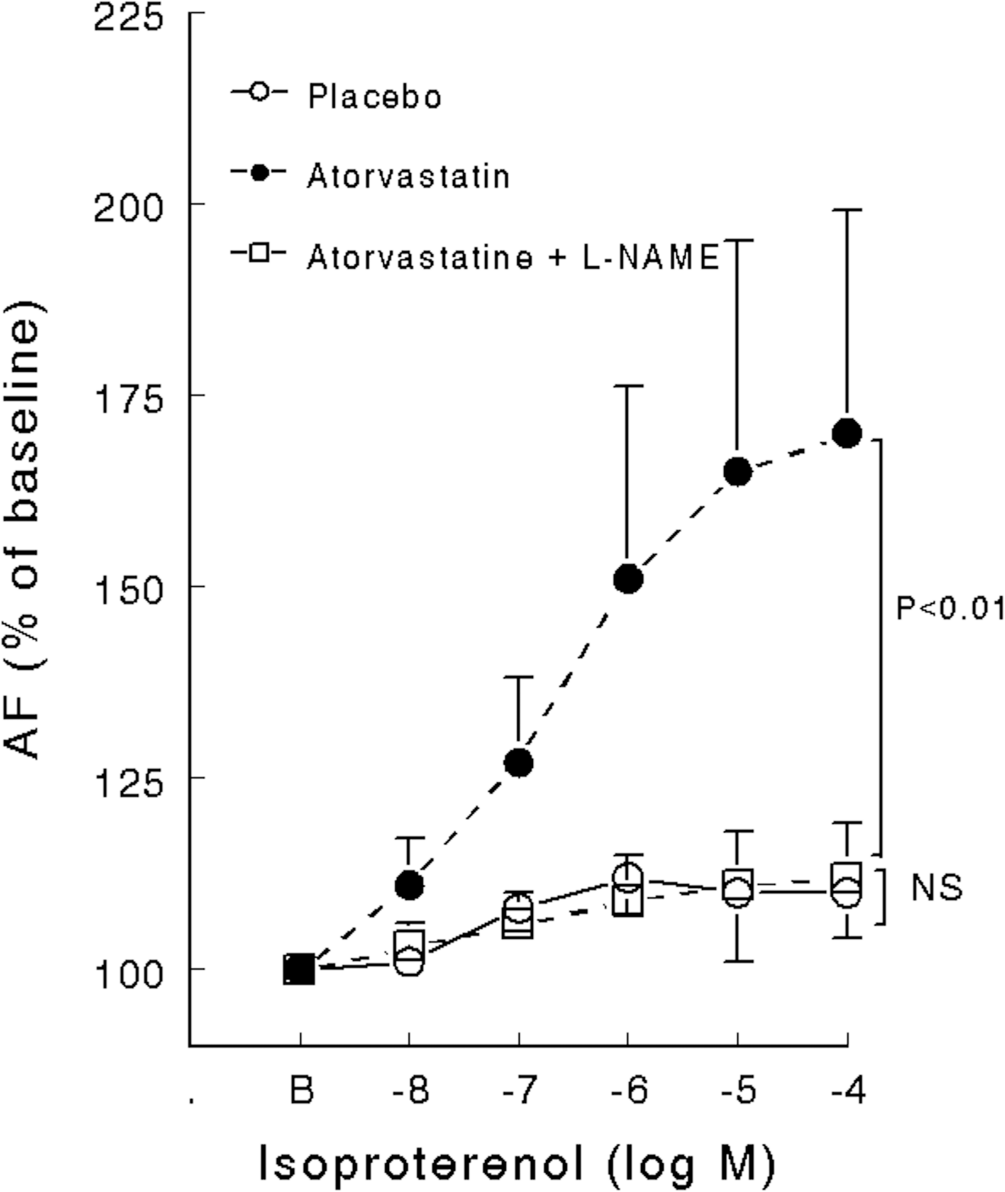
Active force (% of baseline value) variation of left ventricle papillary muscle exposed to isoproterenol in diabetic rats pretreated with statin (atorvastatin, 50 mg kg-1 day-1) during 15 days (8 rats per group) with or without L-NAME administration. Control refers to diabetic rats not receiving statin. AF/s = active force normalized per cross-sectional area during isometric contraction. Data are expressed as mean ± SD. NS: non-significant.

L-NAME, a non-specific NO synthase inhibitor, abolished the beneficial effects of atorvastatin on the β-adrenoceptor response in diabetic left ventricular papillary muscle (Fig 5).

## Discussion

We observed that, in rats with diabetic cardiomyopathy, atorvastatin restored the positive inotropic effect of β-adrenoceptor stimulation whereas it did not correct the basal diastolic dysfunction. Atorvastatin countered the down-regulation of β1-adrenoceptor protein expression and the up-regulation of MRP4 but not that of β3-adrenoceptor. The main mechanism involved in this protective effect was related to NO pathway.

Our results suggest that atorvastatin reduce the β-adrenergic dysfunction in diabetic hearts. The clinical benefits of statins in diabetic patients may be related to the improvement in cardiac adrenergic response, restoring a functional reserve for challenging times such as critically ill conditions or the perioperative period [18]. Catecholamines are widely used in intensive care unit but considerable intra and inter-individual variability exists in the response. Most previous studies have tried to elucidate pharmacokinetic and pharmacodynamic differences in catecholamine response but very few have considered differences linked to the baseline characteristics of the patient such as diabetes and chronic treatment with statins [27].

Diabetic cardiomyopathy is associated with a worsen perioperative prognosis. Apart from changes in basal function and contractile proteins [7], we have previously demonstrated the importance of β-adrenergic dysfunction in diabetic hearts from the dysregulation of β1- and β3-adrenoceptors, the predominance of the NO pathway signaling [6,9]. Inflammation is another key process in diabetic cardiomyopathy, due to hyperglycemia, and increases in levels of free fatty acids and reactive oxygen species [7,10]. In diabetic hearts chronic activation of the PI3K/Akt pro-inflammatory pathway results in more Rho and Ras active proteins and contributes to excitation-contraction coupling abnormalities, myocardial fibrosis, and cardiomyocyte apoptosis [10].

Statins have clinical benefits on morbidity and mortality in a wide range of diseases and particularly in diabetic patients [14,18–19]. Previous experimental studies have explained this positive effect on diabetic hearts by a normalization of RhoA signaling and an increase in NOS3 expression [24,28–29]. Atorvastatin and fluvastatin have been reported to limit the over-expression of pro-inflammatory cytokines, development of cardiac fibrosis and left ventricle remodeling after myocardial infarction in experimental diabetic cardiomyopathy [24, 28–30]. While several clinical trials demonstrated an improved contractility effect of statin in chronic heart failure [15–16], fewer data are available on their effects on cardiac contraction and excitation-contraction coupling in diabetic cardiomyopathy. *In vivo*, statins improve basal LVEF in diabetic cardiomyopathy only when it was reduced on basal measurements [29–31]. *In vitro*, simvastatin improved contraction in neonatal cardiomyocytes of healthy rats [31]. Fluvastatin improved the response of heart rate, arterial pressure and systolic rise of left ventricular pressure to phenylepherine and nitroprusside [32]. Only one study described in neonatal cardiomyocytes a reduction in cAMP elevation in response to isoproterenol after atorvastatin, mediated by down-regulation of αsG-proteins [33]. To our knowledge, although statins have been recently proven to inhibit β-adrenoceptor stimulated apoptosis [34], no consistent data have been published on the impact of atorvastatin on the cardiac response to β-adrenergic stimulation in diabetic cardiomyopathy.

In our study, atorvastatin corrected β-adrenergic cardiac dysfunction in diabetic rats but did not affect basal diastolic dysfunction. As previously reported in diabetic hearts [6], we observed normal systolic function using echocardiography and normal AF in papillary muscles in basal conditions. The positive inotropic response to β-adrenergic stimulation was markedly decreased in diabetic compared to healthy rats as previously described [6]. Prolonged contraction of papillary muscles (increased TPF and TPS) and a diastolic dysfunction (prolonged IVRT) are also common features of the diabetic cardiomyopathy [9]. We observed that atorvastatin partly corrected β-adrenergic dysfunction but did not influence diastolic function in diabetic rats that remained altered compared with healthy rats. A strength of our study is the consistent effects of atorvastatin observed both *in vivo* and *ex vivo*. Atorvastatin did not affect body or heart weight, total and LDL cholesterol or blood glucose level in healthy or diabetic rats, in contrast with previously reported data [32]. These results show that atorvastatin corrected β-adrenergic dysfunction in diabetic rats but did not suppress the features of diabetic cardiomyopathy, strongly suggesting modifications in the β-adrenergic signaling pathway.

In isolated neonatal cardiomyocytes, atorvastatin has been shown to decrease the inotropic response to β-adrenoceptor stimulation via reduced isoprenylation of Gγ protein, without significant changes in β-adrenoceptor density and cAMP production [33]. In adult rat cardiomyocytes, simvastatin potentiated the inotropic response to β2- but not β1-adrenoceptor stimulation [35]. However, in our study, we did not observe any direct effect of atorvastatin on the β-adrenoceptor function in healthy rats.

From the microarray analysis, we evaluated the expression of several proteins involved in the β-adrenergic signaling pathway. As post-transcriptional modifications or microarray biases are possible, we confirmed our results by Western blotting. In our study, atorvastatin improved the β1/β3-adrenoceptor ratio in diabetic hearts contributing to the positive inotropic effect of β-adrenergic stimulation. The expression of β1-adrenoceptor was reduced in diabetic untreated rats compared with healthy rats but corrected by atorvastatin. In contrast, the expression of β3-adrenoceptor increased in both untreated and atorvastatin diabetic rats compared with healthy rats. In diabetic hearts the positive inotropic effect induced by β1-adrenoceptor stimulation is reduced by the down-regulation of β1-adrenoceptor expression [6]. In addition, altered positive inotropic effect is amplified by an increased negative inotropic effect from β3-adrenoceptor stimulation due to β3-adrenoceptor up-regulation [6]. After atorvastatin treatment, the positive inotropic effect from β1-adrenoceptor stimulation was increased by normalization of β1-adrenoceptor expression and counteracts more effectively the β3-adrenoceptor negative inotropic effect even if unchanged. In contrast, statins increase PDE2 RNA production as well, which is known to be involved in ß3-adrenoceptor pathway. Nevertheless, the increase in PDE2 RNA production is probably not enough to limit the benefit of the up-regulation of the ß1-adrenoceptor pathway in treated diabetic rats.

MRP4, known to act as an independent endogenous regulator of cAMP, is upregulated in elderly rats [36–37]. Our study confirmed in diabetic rats. MRP4 overexpression contributes to a decrease in the positive inotropic effect induced by cytosolic cAMP production after β_1_-adrenoceptor stimulations. Our data suggest that atorvastatin decreased the over-expression of MRP4 proteins in treated diabetes, which leads to an increase in the cytosolic cAMP concentration. This decrease in MRP4 expression may be associated with the over-expression of β1-adrenoceptor pathway proteins for off-setting over-expression of PDE2 after atorvastatin treatment. However, we cannot conclude if MRP4 abnormalities observed in diabetes participates to β-adrenoceptor dysfunction or is only a consequence. Anyway, atorvastatin contributes to restore MRP4 protein expression.

The cholesterol-independent, so-called the “pleiotropic” effects of statins seems to be predominantly related to up-regulation and activation of NOS3, mainly through the inhibition of the Rho/ROCK and PI3K/protein kinase Akt pathways, respectively.^23^ The activation of NOS3 by statins occurs through scavenger β1-adrenoceptor, Gi protein, phospholipase C and entry of calcium [38]. More recently, rosuvastatin has been shown to reverse isoproterenol-induced acute myocardial infarction in the rat, at least partly related to up-regulation of NOS2 [39]. We observed that L-NAME, a non-specific NOS inhibitor, abolished the beneficial effects of atorvastatin on the β-adrenoceptor response in diabetic rats (Fig 5). This suggests that the main mechanism involved in the protective effect of statins on the β-adrenoceptor response is also related to either up-regulation and/or activation of NOS. These results are also in agreement with previous results obtained in a non-diabetic rat model of high-fat diet-induced obesity in the rat showing that rosuvastatin restores both adrenergic and nitrergic functions in mesenteric arteries [40]. Statins are known to increase IPK3 activation, which increases NOS3 activity [23]. In parallel, a biphasic concentration-dependent response of myocardial contractility to NO has been shown [41–42]. At low concentrations of NO, a positive inotropic effect is observed while a negative inotropic effect occurs at high concentrations. Because ß-adrenoceptor stimulation induces IPK3 activation [43], results obtained with L-NAME suggest that IPK3-dependant NO production may be involved in the restoration of positive inotropic effect of ß-adrenoceptor stimulation. We did not observe any significant changes in NOS1 or NOS3 but these analyses were performed in whole cardiac muscle and not in isolated cardiomyocytes. We previously showed that NOS1 was involved in altered β-adrenoceptor response in diabetic cardiomyopathy. In contrast, NOS2 and NOS 3 proteins were undetectable in cardiomyocytes [6].

Several points should be taken into consideration in interpreting our results. First, this study was conducted in rat myocardium, which differs from human myocardium. Second, we employed an animal model of type-1 diabetes and we studied only short term effects of diabetes and without insulin treatment. This model requires careful extrapolation to human diabetes, particularly type 2 diabetes although it is considered to be a well-established and reliable model for diabetes mellitus [44]. Third, analysis of gene expression using a microarray has its own limitations related to tissue selection, presentation and interpretation of vast amounts of data. Selected results were confirmed by Western blotting but not for all modified genes. Fourth, the dose of atorvastatin used in the study is higher than in human treatment but is the common regimen used in experimental studies [28]. The effects of other statin drugs have not been evaluated here. Since statins often exhibit class effects, some differences may be expected [45]. Lastly, some concerns have been raised about an increased risk of diabetes onset after high-dose statin treatment [46]. However the benefits of statins in overt diabetes have been widely studied and are not subject to controversy. In contrast, the benefit of statin in critically ill patients remained to be determined [47–48].

In conclusion, we have demonstrated that, in diabetic cardiomyopathy, atorvastatin restores the positive inotropic effect of the β-adrenoceptor stimulation. This effect is the results of a combination of multiple modifications in different steps of β-adrenergic signaling pathway, particularly through the NOS signaling pathway.

## Supporting information

S1 Table Echocardiographic measurement of inotropic and lusitropic response to the β-adrenoceptor stimulation in healthy and diabetic rats pretreated or not with atorvastatin. Data are mean percentages of baseline values ± SD. ^▪^ *p* < 0.05 vs. baseline value; *: *p*<0.05 versus untreated healthy group; †: *p*<0.05 between statin and untreated rats in each group healthy or diabetic rats; ‡: *p*<0.05 between healthy statin rats and diabetic statin rats. T1: baseline; T2: isoproterenol; LVEF = left ventricular ejection fraction; LVSF = left ventricular shortening fraction; IVRT = isovolumic relaxation time; E = peak velocity of early mitral flow; DT = deceleration time of E wave, E/Ea = E peak velocity of early mitral flow/Ea early diastolic velocity of lateral mitral annulus ratio.
(DOCX)

S2 Table Comparison of inotropic response in isotony (Vmax) to β-adrenergic stimulation of left ventricle papillary muscles of healthy or diabetic rats, pretreated or not with atorvastatin. Data are mean ± SD; *: *p*<0.05 versus untreated healthy group; †: *p*<0.05 between statin and untreated rats in each group healthy or diabetic rats; ‡: *p*<0.05 between healthy statin rats and diabetic statin rats. T1: baseline; T2: isoproterenol; V_max_ = maximal unloading isotonic shortening velocity; _max_Eff = maximal effect of isoproterenol on Vmax as percentage of baseline value; C_50_ = concentration of isoproterenol producing 50% of _max_Eff.
(DOCX)

S3 Table Comparison of inotropic response in isometry (AF/s) to β-adrenergic stimulation of left ventricle papillary muscles of healthy or diabetic rats, pretreated or not with atorvastatin. Data are mean ± SD; *: *p*<0.05 versus untreated healthy group; †: *p*<0.05 between statin and untreated rats in each group healthy or diabetic rats; ‡: *p*<0.05 between healthy statin rats and diabetic statin rats. T1: baseline; T2: isoproterenol; AF/s = active force normalized per cross-sectional area during isometric contraction; _max_Eff = maximal effect of isoproterenol on AF as percentage of baseline value; C_50_ = concentration of isoproterenol producing 50% of _max_Eff.
(DOCX)
