# Supplementary material for: Atorvastatin reduces β-Adrenergic dysfunction in rats with diabetic cardiomyopathy
Source: PLoS One. 2017 Jul 20;12(7):e0180103. doi: 10.1371/journal.pone.0180103 (PMC5519044; doi:10.1371/journal.pone.0180103)
Supplement: S1 Table — Data are mean percentages of baseline values ± SD. ▪ p < 0.05 vs. baseline value; *: p<0.05 versus untreated healthy group; †: p<0.05 between statin and untreated rats in each group healthy or diabetic rats; ‡: p<0.05 between healthy statin rats and diabetic statin rats. T1: baseline; T2: isoproterenol; LVEF = left ventricular ejection fraction; LVSF = left ventricular shortening fraction; IVRT = isovolumic relaxation time; E = peak velocity of early mitral flow; DT = deceleration time of E wave, E/Ea = E peak velocity of early mitral flow/Ea early diastolic velocity of lateral mitral annulus ratio. (DOCX) [file pone.0180103.s001.docx]

| **Absolute values** | **Healthy untreated (n= 10)** | | **Healthy statin (n= 10)** | | **Diabetic untreated (n= 9)** | | **Diabetic statin (n = 8)** | |
| --- | --- | --- | --- | --- | --- | --- | --- | --- |
|  | **T1** | **T2** | **T1** | **T2** | **T1** | **T2** | **T1** | **T2** |
| **Heart rate** | 361 ± 35 | 415 ± 31^▪^ | 340 ± 19 | 414 ± 24^▪^ | 301 ± 17 | 384 ± 56^▪^ | 290 ± 31 | 326 ± 22^▪^ |
| **LVEF** | 79 ± 8 | 96 ± 2^▪^ | 79 ± 6 | 95 ± 4^▪^ | 83 ± 6 | 91 ± 5^▪^ | 76 ± 7 | 95 ± 4^▪*^† |
| **LVSF** | 45 ± 6 | 72 ± 7^▪^ | 44 ± 7 | 68 ± 8^▪^ | 48 ± 8 | 58 ± 9^▪^ | 42 ± 7 | 68 ± 9^▪*^† |
| **IVRT** | 15 ± 5 | 14 ± 4 | 15 ± 3 | 9 ± 2 ^▪^† | 25 ± 5 | 20 ± 8 | 29 ± 5 | 25 ± 7 |
| **DT** | 35 ± 7 | 33 ± 4 | 34 ± 6 | 33 ± 7 | 40 ± 12 | 35 ± 6 | 40 ± 15 | 39 ± 11 |
| **E/Ea** | 18 ± 5 | 18 ± 3 | 17 ± 4 | 19 ± 3 | 17 ± 2 | 16 ± 3 | 20 ± 4 | 18 ± 3 |
