# Supplementary material for: Atorvastatin reduces β-Adrenergic dysfunction in rats with diabetic cardiomyopathy
Source: PLoS One. 2017 Jul 20;12(7):e0180103. doi: 10.1371/journal.pone.0180103 (PMC5519044; doi:10.1371/journal.pone.0180103)
Supplement: S2 Table — Data are mean ± SD; *: p<0.05 versus untreated healthy group; †: p<0.05 between statin and untreated rats in each group healthy or diabetic rats; ‡: p<0.05 between healthy statin rats and diabetic statin rats. T1: baseline; T2: isoproterenol; Vmax = maximal unloading isotonic shortening velocity; maxEff = maximal effect of isoproterenol on Vmax as percentage of baseline value; C50 = concentration of isoproterenol producing 50% of maxEff. (DOCX) [file pone.0180103.s002.docx]

| **Isoproterenol** | **Healthy Untreated (n=8)** | | **Healthy Statin (n=8)** | | **Diabetic Untreated rats(n=8)** | | **Diabetic Statin (n=8)** | |
| --- | --- | --- | --- | --- | --- | --- | --- | --- |
|  | **T1** | **T2** | **T1** | **T2** | **T1** | **T2** | **T1** | **T2** |
| **_max_Eff (% baseline value)** | 2.1±0.2 | 3.8±0.4 | 1.7±0.5 | 3.3±0.7 | 2.1±0.3 | 2.7±0.3* | 1.6±0.3 | 2.7±0.4* |
| **C_50_ (µM)** | 0.16±0.15 | 0.20±0.15 | 0.91±1.14 | 0.81±1.15 | 0.07±0.06 | 0.07±0.05 | 0.09±0.08 | 0.28±0.34 |
