# Supplementary material for: Atorvastatin reduces β-Adrenergic dysfunction in rats with diabetic cardiomyopathy
Source: PLoS One. 2017 Jul 20;12(7):e0180103. doi: 10.1371/journal.pone.0180103 (PMC5519044; doi:10.1371/journal.pone.0180103)
Supplement: S3 Table — Data are mean ± SD; *: p<0.05 versus untreated healthy group; †: p<0.05 between statin and untreated rats in each group healthy or diabetic rats; ‡: p<0.05 between healthy statin rats and diabetic statin rats. T1: baseline; T2: isoproterenol; AF/s = active force normalized per cross-sectional area during isometric contraction; maxEff = maximal effect of isoproterenol on AF as percentage of baseline value; C50 = concentration of isoproterenol producing 50% of maxEff. (DOCX) [file pone.0180103.s003.docx]

| **Isoproterenol** | **Healthy untreated (n=8)** | | **Healthy statin (n=8)** | | **Diabetic untreated (n=8)** | | **Diabetic statin (n=8)** | |
| --- | --- | --- | --- | --- | --- | --- | --- | --- |
|  | **T1** | **T2** | **T1** | **T2** | **T1** | **T2** | **T1** | **T2** |
| **_max_Eff (% baseline value)** | 34.4±11.1 | 62.3±19.7 | 32.3±10.6 | 56.1±17.0 | 51.7±12.4 | 57.5±13.1 | 43.4±17.0 | 67.1±28.1 |
| **C_50_ (µM)** | 0.16±0.15 | 0.20±0.15 | 0.91±1.14 | 0.81±1.15 | 0.07±0.06 | 0.07±0.05 | 0.09±0.08 | 0.28±0.34 |
